# Increase of transient lower esophageal sphincter relaxation associated with cascade stomach

**DOI:** 10.3164/jcbn.16-53

**Published:** 2017-04-07

**Authors:** Akiyo Kawada, Motoyasu Kusano, Hiroko Hosaka, Shiko Kuribayashi, Yasuyuki Shimoyama, Osamu Kawamura, Junichi Akiyama, Masanobu Yamada, Masako Akuzawa

**Affiliations:** 1 Department of Medicine and Molecular Science, Gunma University Graduate School of Medicine, 3-39-15 Showamachi, Maebashi-shi, Gunma 371-8511, Japan; 2 Department of Endoscopy and Endoscopic Surgery, Gunma University Hospital, 3-39-15 Showamachi, Maebashi-shi, Gunma 371-8511, Japan; 3 Hidaka Hospital, 886 Nakaomachi, Takasaki-shi, Gunma 370-0001, Japan

**Keywords:** cascade stomach, esophageal manometry, gastric emptying, gastroesophageal reflux disease, liquid test meal

## Abstract

We previously reported that cascade stomach was associated with reflux symptoms and esophagitis. Delayed gastric emptying has been believed to initiate transient lower esophageal sphincter relaxation (TLESR). We hypothesized that cascade stomach may be associated with frequent TLESR with delayed gastric emptying. Eleven subjects with cascade stomach and 11 subjects without cascade stomach were enrolled. Postprandial gastroesophageal manometry and gastric emptying using a continuous ^13^C breath system were measured simultaneously after a liquid test meal. TLESR events were counted in early period (0–60 min), late period (60–120 min), and total monitoring period. Three parameters of gastric emptying were calculated: the half emptying time, lag time, and gastric emptying coefficient. The median frequency of TLESR events in the cascade stomach and non-cascade stomach groups was 6.0 (median), 4.6 (interquartile range) vs 5.0, 3.0 in the early period, 5.0, 3.2 vs 3.0, 1.8 in the late period, and 10.0, 6.2 vs 8.0, 5.0 in the total monitoring period. TLESR events were significantly more frequent in the cascade stomach group during the late and total monitoring periods. In contrast, gastric emptying parameters showed no significant differences between the two groups. We concluded that TLESR events were significantly more frequent in persons with cascade stomach without delayed gastric emptying.

## Introduction

Cascade stomach (CS) is the term for a stomach with retroflexion of the fundus.^(1)^ CS has generally been regarded as a variant of the normal stomach with no pathological significance. On the other hand, ingested food and fluid or swallowed air will initially pool in the fundus of a person with CS. Gastric acid is mainly secreted from the upper body and fundus of the stomach, so acid could also pool in the fundus of a person with CS. Moreover, we previously reported that people with CS have a high incidence of reflux symptoms, as well as dyspepsia,^(2)^ and we recently demonstrated an association between CS and endoscopic reflux esophagitis.^(3)^ It is well known that transient lower esophageal sphincter relaxation (TLESR) is a major mechanism of gastroesophageal reflux (GER).^(4–8)^ Several reports have indicated that delayed gastric emptying might promote gastric distention and increase the frequency of TLESR in humans.^(9–16)^ In patients with non-erosive reflux disease (NERD), TLESR is also the principal mechanism underlying reflux events.^(17)^ We reported that patients with NERD are suffered from not only reflux symptoms but also dyspeptic symptoms.^(18)^ Moreover, in the Japanese guidelines for functional dyspepsia,^(19)^ it is stated that multiple factors (such as gastro-intestinal motility and autonomic nervous system),^(20)^ including morphology of the stomach (cascade stomach) may be associated with the pathophysiology of functional dyspepsia. However, precise pathophysiology of cascade stomach has not been investigated by now. With regard to the possible pathophysiologic effects of CS, we can consider two mechanisms: 1) delayed gastric emptying due to pooling of food/fluid or gastric juice in the fundus and 2) direct stimulation by distension of the gastric fundus.

We hypothesized that persons with CS could have frequent TLESR due to modification of gastric emptying. Therefore, we simultaneously evaluated postprandial TLESR by esophagogastric manometry and assessed gastric emptying by a ^13^C breath test to investigate the relationship between TLESR and gastric emptying in persons with CS.

## Materials and Methods

### Subjects

From March 2013 to March 2014, 22 healthy volunteers were recruited prospectively when they underwent a health-screening barium study of the stomach at Hidaka Hospital, which is affiliated with Gunma University Hospital. Eleven subjects (9 men and 2 women) had CS and 11 non-CS subjects (7 men and 4 women) were also enrolled as a control group (Table 1). None of the subjects were taking antacids, including proton pump inhibitors or histamine H_2_ receptor antagonists, or medications that could influence gastrointestinal motility, and none of them had a history of gastrointestinal disease or surgery. The study protocol was approved by the Ethics Committee of Gunma University Hospital (#870) and written informed consent was obtained from all subjects.

### Diagnosis of cascade stomach

Barium studies were performed with 165 ml of 180 w/v% barium sulfate (Kaigen, Co., Tokyo, Japan) and 6 g of effervescent salts (Kaigen). To prevent gastric contraction, scopolamine butylbromide (20 mg i.m.) was administered, except in subjects >65 years old; those with a history of heart disease, diabetes mellitus, glaucoma, or prostate disease; and those who refused it. All radiographic data were stored in a digital recording system (Digital Radiography DSTATION ARD-100A, Toshiba, Co., Tokyo, Japan), and were displayed on a viewer (519 × 519 pixels, Toshiba) for reading. After esophagography, the first filling view of the stomach containing the entire 165 ml of barium was used to classify gastric morphology.

**Fig. 1:**
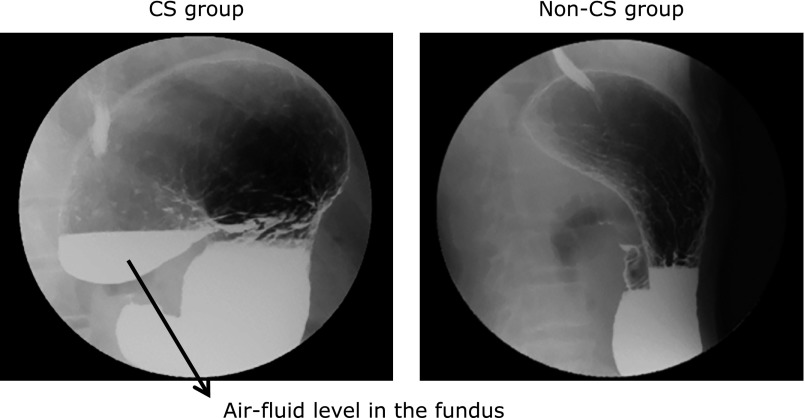
Classification of the stomach on X-ray films. CS was defined by detection of an air-fluid level in the fundus on an upright barium X-ray film. CS, cascade stomach; non-CS, non-cascade stomach.

CS was defined as being present if an air-fluid level was seen in the fundus on an upright barium X-ray film (Fig. 1).

### Test meal and assessment of gastric emptying

The gastric emptying rate was determined by performing a continuous ^13^C acetate breath test using the Breath ID system (Breath ID, Exalenz Bioscience Ltd., Israel) and Breath ID software for data analysis. Breath samples were automatically obtained continuously through a nasal cannula for 120 min after each subject drank a liquid test meal simultaneously with the performance of esophageal manometry.

The liquid test meal was 250 ml of Ensure H^®^ (Abbott Japan, Co., Ltd., Tokyo, Japan), which provided 375 kcal of energy, including 13.2 g of protein, 13.2 g of fat (31.5%), and 51.5 g of carbonate. Each subject drank the test meal through a straw within 5 min (50 ml/min).

### Measurement of esophageal motility

Esophageal manometry was performed with a high-resolution manometry catheter (ManoScan A HRM) with 36 circumferential solid-state sensors set at 1-cm intervals (Given Imaging, Ltd., Yoqneam, Israel), and data were analyzed using Mano view ver. 2.0.1 software. With the subject in a semi-sitting position, the catheter (outer diameter: 4.2 mm) was inserted trans-nasally after anesthesia with 2% viscous lidocaine hydrochloride (AstraZeneca, Tokyo, Japan), and was positioned with 2–3 sensors in the stomach to record data from the pharynx to the esophagogastric junction (EGJ).

The pressure transducers were calibrated at 0 and 300 mmHg before each examination. After adaptation for 5 min, baseline esophageal manometry data were recorded for 30 min before the test meal. After 20 to 30 min of recording, the mean EGJ pressure was also measured over 30 s without swallowing, and the mean basal EGJ pressure and EGJ length were calculated.

### Definition of TLESR

**Fig. 2:**
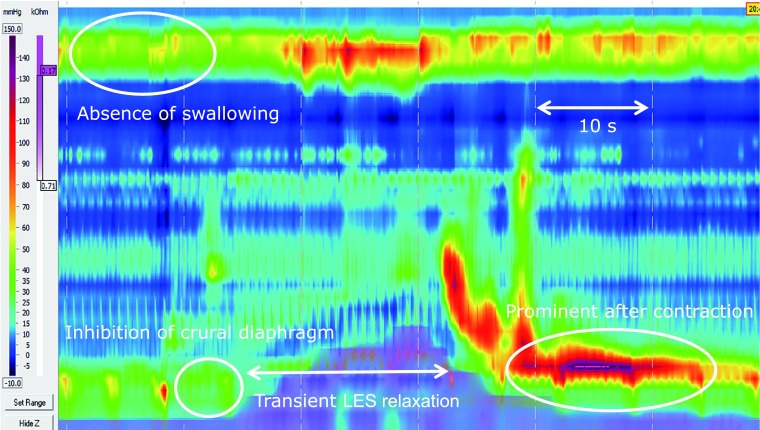
Example of transient lower esophageal sphincter relaxation. LES, lower esopahgeal sphincter.

Based on the report by Roman^(21)^ and Holloway,^(22)^ TLESR were identified by 3 of authors (AK, JA and SK) as contractions meeting at least four of the following six criteria: (1) no swallowing from 4 s before to 2 s after the onset of relaxation, (2) LES relaxation at ≥1 mmHg/s, (3) time of ≤10 s from onset to complete relaxation, (4) nadir pressure ≤2 mmHg, (5) inhibition of the crural diaphragm, and (6) prominent after-contraction (Fig. 2). The duration of each contraction was also determined.

### Data collection

As parameters of gastric emptying, the half emptying time (T1/2), lag time (T lag), and gastric emptying coefficient (GEC), were calculated over 2 h based on the methods reported by Ghoos *et al.*^(23)^ To assess gastric emptying in detail, the percentage of the ^13^CO_2_ dose in the test meal emptied per h (%dose/h) was evaluated every 15 min. The number of TLESR events was counted in the early monitoring period (0–60 min), the late monitoring period (60–120 min), and the total monitoring period (2 h). The mean basal EGJ pressure and the EGJ length were expressed in mmHg and cm, respectively.

### Statistical analysis

TLESR and gastric emptying parameters (T1/2, T lag, and GEC) are expressed as the median with interquartile range (IQR). The difference in the frequency of TLESR events between the CS and non-CS groups and the differences of gastric emptying parameters were analyzed by Wilcoxon’s signed-rank test. The %dose/h values determined in the ^13^C breath test, BMI, age, and TLESR duration are expressed as the mean ± SD, and were analyzed by Welch’s Student *t* test. All statistical analyses were performed with SPSS software (ver. 10). Differences were accepted as significant at *p*<0.05.

## Results

**Fig. 3:**
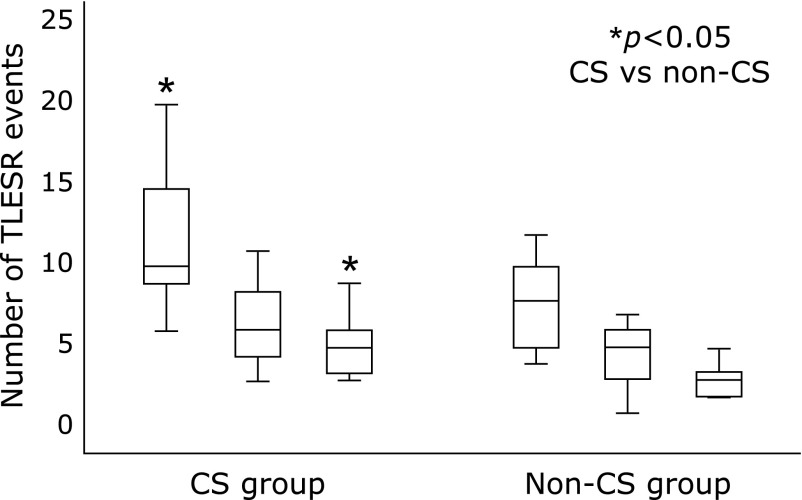
Comparison of the number of TLESR events. During the late period and, total period, transient lower esophageal sphincter relaxation (TLESR) events were significantly more frequent in the CS group than in the non-CS group. TLESR events were also more frequent during the early period in the CS group than in the non-CS group, but there was no significant difference. CS, cascade stomach; TLESR, transient lower esophageal sphincter relaxation; TP, total monitoring period; EP, early monitoring period; LP, late monitoring period, *p<0.05.

Demographic characteristics and EGJ parameters of the CS and non-CS groups are shown in Table 1. There were no significant differences of age and BMI between the CS and non-CS groups. There were also no significant differences of the mean basal EGJ pressure and EGJ length between the two groups. A total of 134 TLESR events were detected in the CS group and 85 events were detected in the non-CS group. TLESR events (median, IQR) were significantly more frequent in the CS group than in the non-CS group during the late monitoring period (5.0, 3.2 vs 3.0, 1.8) and the total monitoring period (10.0, 6.2 vs 8.0, 5.0). During the early monitoring period, the frequency of TLESR was also higher in the CS group than in the non-CS group (6.0, 4.6 vs 5.0, 3.0), but there was no significant difference. The duration of TLESR events showed no significant difference between the two groups in any of the periods (Fig. 3). Gastric emptying parameters of the two groups are displayed in Table 2.

**Fig. 4:**
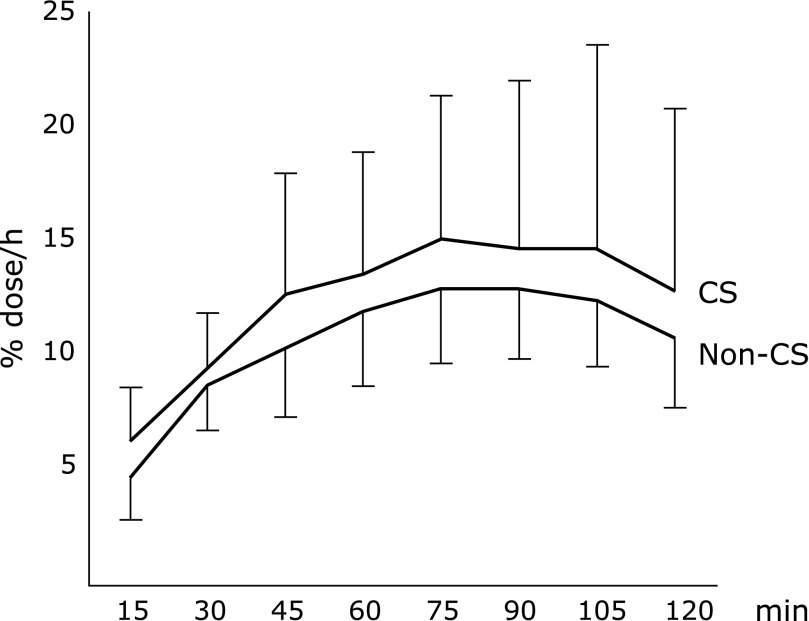
Results of the breath test. Continuous^13^CO_2_ excretion (%dose/h) in the two groups. Values are the mean excretion of ^13^CO_2_ in the CS group and the non-CS group (mean ± SD).

There were no significant differences between the two groups. The %dose/h values measured every 15 min also showed no significant differences between the two groups (Fig. 4).

## Discussion

The present study clearly demonstrated an increased frequency of TLESR events in persons with CS after ingestion of a liquid test meal. We also found that delayed gastric emptying was not responsible for the initiation of TLESR in our subjects with CS. Gastroesophageal reflux disease (GERD) patients have a significantly increased frequency of TLESR events associated with acid reflux in Western countries,^(5,7,8,17)^ and also in Japan.^(18)^ TLESR is triggered by sensory signals from receptors in the proximal stomach (especially the subcardiac region), esophageal body, and pharynx. These signals are integrated in brain stem and trigger a motor response that involves LES relaxation and inhibition of the crural muscles. Several reports have indicated that prolonged gastric distention or delayed gastric emptying associated with gastric distention might increase TLESR in humans.^(12–16)^ It has also been reported that an increase of postprandial TLESR is correlated with BMI,^(4,24)^ but the reasons for an increased frequency of postprandial TLESR in obese persons are still incompletely understood.

We previously reported that persons with CS had a high incidence of reflux symptoms as well as dyspepsia.^(2)^ More recently, we reported that CS was a risk factor for developing endoscopic reflux esophagitis.^(3)^ Our studies suggest that administration of proton pump inhibitor could be effective not only for persons with reflux esophagitis,^(25)^ but also for person with CS and NERD.^(26)^ CS is defined as retroflexion of the gastric fundus and is thought to occur because of gas in the transverse colon^(1)^ or other organs, operative adhesions^(27)^ and cancer of the pancreas.

In addition, we reported that visceral fat related to obesity or metabolic syndrome could be an important cause of CS.^(2)^ A positive association between obesity and GERD has been reported in many epidemical or cross-sectional studies.^(28–31)^

Our previous study performed in a large number of subjects revealed that BMI was higher in persons with CS than those without CS.^(2)^ To avoid the influence of obesity in the present study, we matched the BMI of the CS and non-CS groups, so a difference of obesity and intra-abdominal pressure could not explain the increased frequency of TLSER events in the CS group. Also, all subjects ingested the same test meal within a 5-min period, thus excluding any influence of differences in the volume or speed of intake. Furthermore, we excluded the effect of swallowed air because all subjects drank the liquid test meal at same speed (50 ml/min) through a straw. The gastric emptying curves of our two groups showed no differences, suggesting that the higher frequency of TLESR events in the CS group was not due to delayed gastric emptying but was related to gastric distension caused by the anatomy of CS. We also measured EGJ pressure, EGJ length, and the duration of TLESR in order to investigate the influence of EGJ function on TLESR, but there were no significant differences of these parameters between the two groups.

Some limitations of this study need to be considered. First, we did not employ a pH sensor to evaluate the acidity of GER because we only inserted a motility sensor in order to maintain sufficient airway to continuously sample expired air from a nasal cannula for the breath test. Another limitation was use of a liquid test meal. If the subjects had been given a high-fat solid meal, we might have observed a difference of gastric emptying between the two groups.

In conclusion, postprandial TLESR events showed a significantly higher frequency in the CS group than in the non-CS group. This difference of TLESR was not caused by delayed gastric emptying, but could have arisen from distention of the gastric fundus related to the anatomical characteristics of CS. These findings suggest that CS is the one of the factors predisposing patients to the development of GERD.

## Figures and Tables

**Table 1:** Comparison of demographic data, EGJ parameters, and duration of TLESR

	CS group	Non-CS group	*p* value
Age (mean ± SD) (years)	34.3 ± 13.3	34.9 ± 12.3	ns
BM I (mean ± SD)	22.3 ± 3.5	20.5 ± 1.7	ns
Duration of TLESR (mean ± SD) (s)	18.7 ± 4.2	20.2 ± 5.5	ns
EGJ length (mean ± SD) (cm)	3.72 ± 0.41	3.78 ± 0.82	ns
EGJ pressure (mean ± SD) (mmHg)	20.3 ± 11.5	15.9 ± 9.6	ns

*P* values were determined by student’s *t* test (Welch). SD, standard deviation; ns, not significant; CS, cascade stomach; non-CS, non cascade stomach; EGJ, esophagogastric junction; TLESR, transient lower esophageal sphincter relaxation.

**Table 2:** Comparison of gastric emptying parameters

	CS group	Non-CS group	*p* value
T 1/2 (Median, IQR) (min)	130.0, 82.3	120.6, 33.4	ns
T lag (Median, IQR) (min)	74.2, 30.6	72.5, 20.6	ns
GEC (Median, IQR) (%dose/h)	3.33, 0.70	3.42, 1.1	ns

*P* values were determined by the Wilcoxon signed-rank test. There were no significant (ns) differences between the two groups. CS, cascade stomach; non-CS, non cascade stomach; T 1/2, half empting time; T lag, excretory lag phase; GEC, gastric empting coefficient; IQR, interquartile range.

## Abbreviations

CS: cascade stomach
EGJ: esophagogastric junction
GEC: gastric empting coefficient
GERD: gastroesophageal reflux disease
IQR: interquartile range
NERD: non-erosive reflux disease
TLESR: transient lower esophageal sphincter relaxation
